# A Multicenter Single-Arm Objective Performance Criteria Trial to Determine the Efficacy and Safety of High-Frequency Irreversible Electroporation as Primary Treatment for Localized Prostate Cancer: A Study Protocol

**DOI:** 10.3389/fonc.2021.760003

**Published:** 2021-11-10

**Authors:** Bi-Ming He, Wei Xue, Wei-Gang Yan, Lei Yin, Bai-Jun Dong, Zhi-En Zhou, Heng-Zhi Lin, Yi Zhou, Yan-Qing Wang, Zhen-Kai Shi, Hai Zhou, Shuai-Dong Wang, Shan-Cheng Ren, Xu Gao, Lin-hui Wang, Chuan-Liang Xu, Hai-Feng Wang

**Affiliations:** ^1^ Department of Urology, Shanghai East Hospital, School of Medicine, Tongji University, Shanghai, China; ^2^ Department of Urology, Renji Hospital, School of Medicine, Shanghai Jiaotong University, Shanghai, China; ^3^ Department of Urology, Peking Union Medical College Hospital, Chinese Academy of Medical Sciences, Beijing, China; ^4^ Department of Urology, Changzheng Hospital, Second Military Medical University, Shanghai, China; ^5^ Department of Urology, Changhai Hospital, Second Military Medical University, Shanghai, China

**Keywords:** H-FIRE = High-frequency irreversible electroporation, prostate cancer, multicenter analysis, clinical trial, protocol

## Abstract

**Introduction:**

The classical pathway for the therapy of low- to intermediate-risk localized prostate cancer is radical prostatectomy or radiation therapy, which has shown a high incidence of complications, including erectile dysfunction, urinary incontinence, and bowel injury. An alternative pathway is to perform an ablation by some energy to the localized lesion, known as focal therapy. High-frequency irreversible electroporation (H-FIRE) is nonthermal energy that can be used in cancer ablation to deliver pulsed high-voltage but low-energy electric current to the cell membrane and to invoke cell death. An H-FIRE pathway has been reported to be tissue-selective, which leads to fewer side effects.

**Methods and Analysis:**

This is a multicenter and single-arm objective performance criteria (OPC) study, in which all men with localized prostate cancer are allocated to H-FIRE ablation. This trial will assess the efficacy and safety of the H-FIRE ablation for prostate cancer. Efficacy will be assessed by prostate biopsy 6 months after treatment while safety will be assessed by adverse event reports and questionnaires. The main inclusion criteria are moderate to low-risk prostate cancer in NCCN risk classification and had no previous therapy for prostate cancer. A sample size of 110 participants is required. The primary objective is to determine whether the detection rate of clinically significant cancer by prostate biopsy is less than 20% after the H-FIRE ablation.

**Ethics and Dissemination:**

This study has obtained ethical approval by the ethics committee of all participating centers. The results of the study will be submitted for dissemination and publication in peer-reviewed journals.

**Conclusions:**

This multicenter single-arm objective performance criteria trial will evaluate the efficacy and safety of the use of high-frequency irreversible electroporation in treating prostate cancer.

**Strengths and Limitations of This Study:**

A comprehensive evaluation of imaging and histopathology is used to determine the effect of treatment. Questionnaires were used to assess the treatment side effects. Multicenter and pragmatic designs capacitate higher generalizability. A limitation of this trial is that the prostate biopsy as an endpoint may not be as accurate as of the specimen from prostate prostatectomy. Another limitation is the 6-month follow-up time, making this trial challenging to come to firm conclusions regarding the efficacy and safety of IRE in the long term.

**Clinical Trial Registration:**

ClinicalTrials.gov, NCT03838432

## Introduction

Prostate cancer is the second most prevalent male cancer and the fifth most common cancer death for men worldwide, which accounted for 13.5% of cancer cases and 6.7% of cancer deaths (1). The traditional pathway for the therapy of low to intermediate-risk prostate cancer, radical prostatectomy, or radiation therapy aims to eliminate the whole gland rather than the localized cancer tissue. Although the treatment is efficacy, it may damage the neurovascular bundles, bladder neck, and rectum, which will lead to complications of erectile dysfunction, urinary incontinence, and bowel injury. These complications and side effects seriously reduce the quality of life. Another pathway in treating low-risk disease is active surveillance, also to prove the efficacy and safety (2). However, there are several barriers, such as patient education or anxiety that were challenging for the continuation of active surveillance (3). Also, it will bring psychosocial and financial burdens for patients who were under active surveillance for a long period of time.

To maintain the efficacy and avoid the side effect of the current pathway, a selective pathway between active surveillance and radical treatment is emerging, which is known as focal therapy. This pathway aims to destroy the focal cancer lesions and preserve the surrounding benign tissue. There are various energy sources that have been used for focal therapy. The prevalent type of energy is thermal energy, including high-intensity focused ultrasound (HIFU), laser, and cryotherapy, which lead to lethal effects by generating extreme temperatures (4). Another novel nonthermal technique for ablation is irreversible electroporation (IRE), which delivers pulsed high voltage, but the low-energy direct electric current through the specialized electrode needles generates a short and intense electrical field to the cell membrane, thereby invoking cell death (5). The potential advantage of IRE seems to be tissue-selective with little damage to the connective tissue structure (6). That is to say, some vital structures like blood vessels or nerves may be reserved after an ablation procedure (6, 7). The traditional IRE applies 80 to 120 unipolar pulses with a pulse of 50 to 100 μs and an electric field >1,000 V/cm. However, this typical IRE protocol may evoke muscle contraction during the procedure, leading to pain for the patients and causing displacement of the electrode needles. Recently, a new type of IRE technique called high-frequency IRE (H-FIRE) has emerged (8). H-FIRE utilizes a set of bipolar pulse burst. Each burst consists of some individual pulses range from 0.5 to 10 μs, and the total time of a single burst is up to 100 μs. Some animal experiments had verified the efficacy of H-FIRE in tumor killing and reduction in muscle contraction. We also preliminarily confirmed that H-FIRE could preserve the urethra and major vasculature in the prostate in the human pilot trial (9).

This multicenter trial aims to evaluate the efficacy and safety of H-FIRE ablation for men with localized prostate cancer. The primary objective is to determine if the clinically significant cancer is detected by less than 20% by prostate biopsy after the ablation.

### Outcomes

The primary outcome is the detection rate of clinically significant prostate cancer, defined as any biopsy core with a Gleason score of 3 + 4 or more, or a Gleason score of 3 + 3 plus maximum cancer core length at >3 mm, or an increase from the original cancer burden in the ablation area 6 months post H-FIRE.

The main secondary objectives are as follows:

1. To assess the proportion of men with serum PSA level at >2 ng/ml over the nadir in 6 months post H-FIRE;
2. To assess the adverse events according to Common Terminology Criteria for Adverse Events (CTCAE) at any time post H-FIRE;
3. To assess the proportion of men using pads preoperative and 1 months, 3 months, and 6 months post H-FIRE;
4. To assess the urinary symptoms by questionnaire of International Prostate Symptom Score (IPSS) scoring preoperative and 1 months, 3 months, and 6 months post H-FIRE;
5. To assess the erectile symptoms by International Index of Erectile Function (IIEF-5) scoring preoperative and 1 months, 3 months, and 6 months post H-FIRE; and
6. To assess the time of urinary catheter retention.

### Trial Design

**Figure 1:**
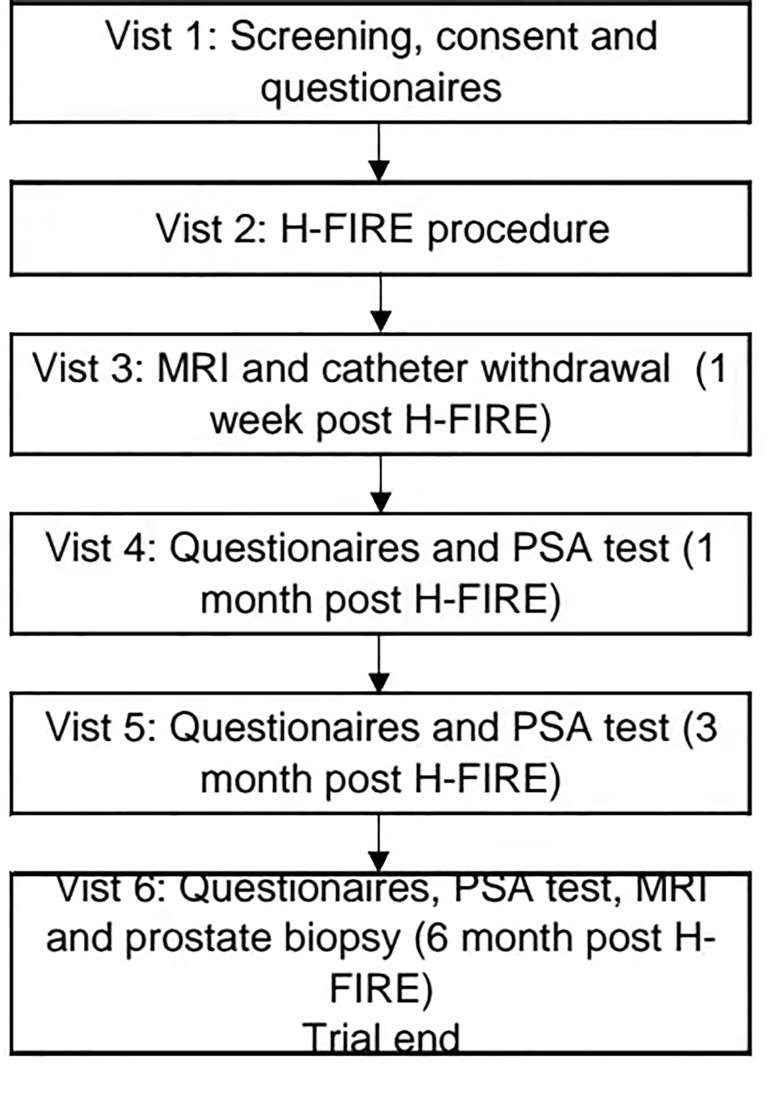
Trial flowchart.

This prospective, multicenter and single-arm objective performance criteria (OPC) trial will take place at four academic medical centers in China: Changhai Hospital, Shanghai; Renji Hospital, Shanghai; Peking Union Medical College Hospital, Beijing; and Changzheng Hospital, Shanghai. The main objective of this study is to assess the efficacy and safety of high-frequency irreversible electroporation (H-FIRE) for the ablation of prostate cancer. All participants who meet the entry criteria will undergo H-FIRE and subsequently receive prostate biopsy 6 months later (**Figure 1**). This trial is approved by the committee for medical and health ethics of all centers and registered on ClinicalTrials.gov (NCT03838432).

## Methods and Analysis

### Patient Population

Patients with a new diagnosis of low-risk or intermediate-risk prostate cancer in NCCN risk classification and who had no previous therapy for prostate cancer will be seen as qualifying for registration in the trial if they can meet all inclusion criteria and had no exclusion criteria. The main criteria include men with elevated serum PSA less than 20 ng/ml, suspected stage less than or equal to T2c, and biopsy Gleason score less than or equal to 4 + 3. The details of inclusion and exclusion criteria are shown in **Table 1**. All eligible patients will be informed in detail; only those who sign the consent will enter the trial. Patients who are ineligible or do not want to participate in this trial will be returned to the regular clinical pathway, active surveillance, or radical treatment.

**Table 1:** Patients inclusion and exclusion criteria.

Inclusion criteria
Age range from 40 to 85 years old
Prostate cancer must be detected by template-guided mapping biopsy or targeted plus template-guided mapping biopsy
Serum PSA < 20 ng/ml
Suspected stage ≤ T2c (organ confined)
Biopsy Gleason score ≤ 4 + 3
No prostatic calculus or prostatic calculus ≤ 5 mm
Fully understand the clinical trial protocol and sign the informed consent
**Exclusion criteria**
Previous history of radical prostatectomy, hormonal therapy, or radiotherapy
Previous surgery within 3 months
Long-term medication with anticoagulant or stop taking anticoagulant less than 1 months
Not fit for anesthesia or surgery
With cardiac pacemaker and/or with metal implants which are located in the range from the first lumbar vertebra (L1) to the middle of femurs.
Previous history of epilepsy
Any other malignant tumor or HIV

### Baseline Characteristics

Blood and urine samples will be collected before IRE for a routine examination. Performance status (Eastern Cooperative Oncology Group, ECOG) will be used to evaluate the general health status and tolerance to treatment. Also, the questionnaire of International Prostate Symptom Score (I-PSS) will be used to assess the baseline urinary symptoms, and the International Index of Erectile Function (IIEF-5) will be used to evaluate the baseline erectile symptoms (10).

### Multi-Parametric Magnetic Resonance Imaging

All participants who sign the informed consent will undergo a 3.0-T multiparametric magnetic resonance imaging (mpMRI) three times: within 1 month pre H-FIRE, 1 month post H-FIRE, and 6 months post H-FIRE. The sequences of examination mainly included T2-weighted imaging (T2WI), diffusion-weighted imaging (DWI), and dynamic contrast-enhanced imaging. Images will be evaluated and scored by expert radiologists according to the Prostate Imaging Reporting and Data System (PI-RADS) (11). The MRI before H-FIRE aims to be performed for localizing the tumor lesions and for scheming the ablation region or range. After the MRI examination, patients will undergo a 12–20-region template-guided prostate biopsy combined with targeted biopsy (for MRI-positive patients) to locate the lesions of the prostate (12, 13). The coordinate of each biopsy core will be marked down. Hence, we can target the focal lesion precisely according to the coordinate. The follow-up MRI will reevaluate the prostate, both the ablation area and untreated area, and provide the evidence for the prostate biopsy 6 months post H-FIRE.

### Clinical Pathway

Participants will be hospitalized 1 day before the IRE. The IRE will be performed *via* the perineum with the guidance of ultrasound under general anesthesia. A Foley catheter and prophylactic antibiotics will be given to patients after the procedure. All participants will be scheduled to be dismissed the following day after IRE. After discharge, the patient will take 1 week of antibiotics, alpha1-adrenoceptor antagonists, and painkillers. The catheter will be retained for 3 to 14 days according to the ablation range and area and will be withdrawn at the outpatient clinic. If the patient is unable to void after removing the catheter, he will be given another Foley catheter for 1 more week.

### Follow-Up and Study Endpoint

MRI will be organized 1 and 6 months after IRE, and serum PSA measurement will be organized 1, 3, and 6 months. Patients will be scheduled to respond the questionnaires including I-PSS, IIEF-5, and pad usage records at 1, 3, and 6 months. At 6 months, all participants will receive a prostate biopsy to get the sample for pathological evaluation. The details and timeframe of follow-up are shown in **Table 2**. This trial will discontinue due to the following: (1) more than 20% of the total number of cases deviated from the protocol; (2) the number of dropout cases exceeds 20% of the total number of designed cases; (3) the severity and frequency of serious adverse events that occurred tended to exceed those reported in local treatment literature; and (4) the severity and frequency of instrument defects and faults exceed the acceptable risk.

**Table 2:** Participant timeline in the study.

	Contact with patient					
	Vist 1	Vist 2	Vist 3	Vist 4	Vist 5	Vist 6
	Pre H-FIRE	H-FIRE	1-week post H-FIRE	1-month post H-FIRE	3-month post H-FIRE	6-month post H-FIRE
Time	-30~0 days	0	7~10 days	4~6 weeks	12~14 weeks	6~7 months
Consent	×					
Screening	×					
Baseline characteristic	×					
PSA	×			×	×	×
Routine blood test	×		×	×	×	×
Routine urine test	×		×	×	×	×
Blood biochemistry test	×		×	×	×	×
ECT	×					
Performance status	×					
MRI	×		×			×
Prostate biopsy	×					×
Pathological assessment	×					×
NCCN risk assessment	×					
Clinical stage assessment	×					
Pad usage record	×			×	×	×
IIEF–5 scoring questionnaire	×			×	×	×
IPSS scoring questionnaire	×			×	×	×
Urinary catheter removed			×			
Withdrawal	Complete as required at any time following registration					
SAE	Complete as required at any time following registration					

### Prostate Biopsy

All patients will undergo ultrasound-guided transperineal prostate biopsy when the 6-month post H-FIRE MRI report is deposited to get the sample of the prostate for assessing the efficiency of H-FIRE. We will sample both the ablation region and the untreated area to minimize missed tumors. The combined biopsy will be performed under local anesthesia with a two- or three-step procedure after reviewing the MRI image and report. Firstly, a cognitive fusion-targeted biopsy will be taken with three cores for each ablation area. Secondly, another targeted biopsy with three cores for each suspicious lesion will be taken only when there is some finding with a PI-RADS score more or equal to 3 on the MRI (13). Lastly, a 12- to 20-region systematic template-guided biopsy will follow (12). The template-guided biopsy region will be a little different between the centers but will be the same as the pre H-FIRE biopsy and the same within the same center.

### Calculation of Sample Size

The detection rate of significant cancer after focal therapy is clinically acceptable to be 20% (14). The previous data from the Changhai hospital showed the detection rate of significant cancer after ablation to be 9%.

For the single arm OPC hypothesis, employing 80% power, 2.5% one-sided α, and a 95% two-sided confidence interval, 87 men will be required. Assuming there is a 20% withdrawal/loss in follow-up, 110 participants are expected to be enrolled.

### Statistical Methods

All statistical tests will use a two-sided test. p values less than or equal to 0.05 will be considered statistically significant (except for special instructions). In addition to point estimation, 95% confidence intervals will be calculated for all results. All continuous variables will be described using the mean, standard deviation, median, minimum, maximum, first quartile (Q1), and third quartile (Q3), and categorical variables will be described using frequencies and proportions. Appropriate methods will be used to analyze the comparison according to the types of variables. Student’s t test or Wilcoxon signed-rank test will be used for the comparison of quantitative data between groups; chi-square test or Fisher’s exact test will be used for categorical data; and Wilcoxon signed rank test or Cochran–Mantel–Haenszel (CMH) test will be used for ranked data.

### Irreversible Electroporation Strategy and Procedure

**Figure 2:**
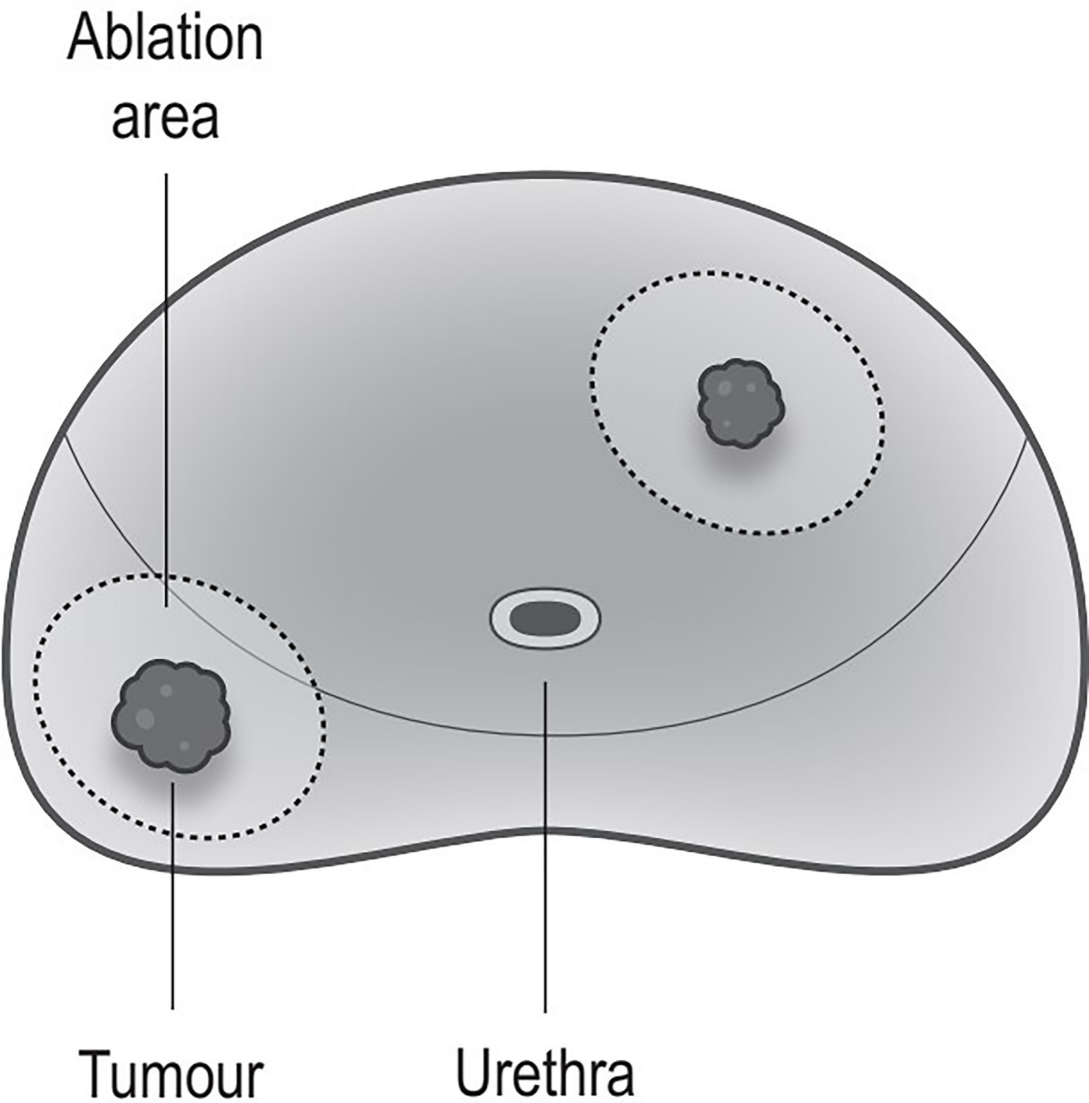
Ablation strategy. Ablation every lesions; every ablation area includes the tumor and some surrounding benign tissues (the safety margin is >1 cm).

**Figure 3:**
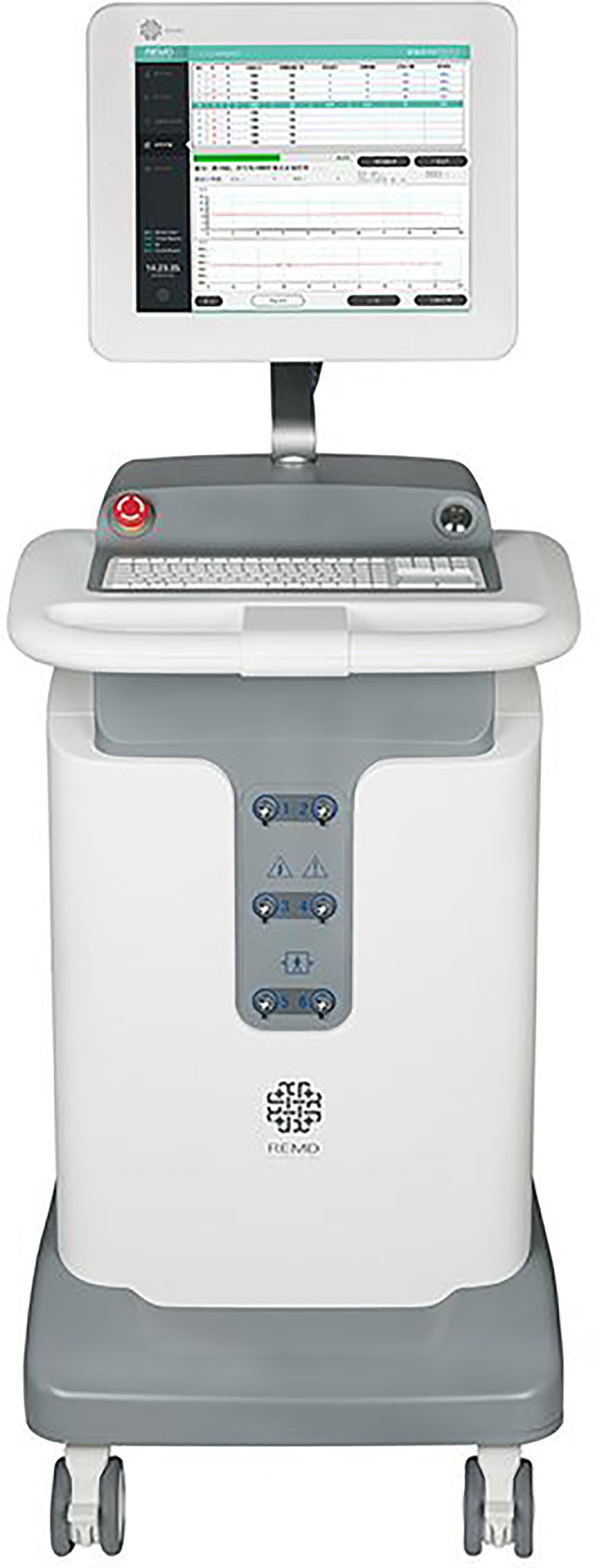
The composite steep pulse therapeutic apparatus (REMEDINE, Shanghai, China).

**Figure 4:**
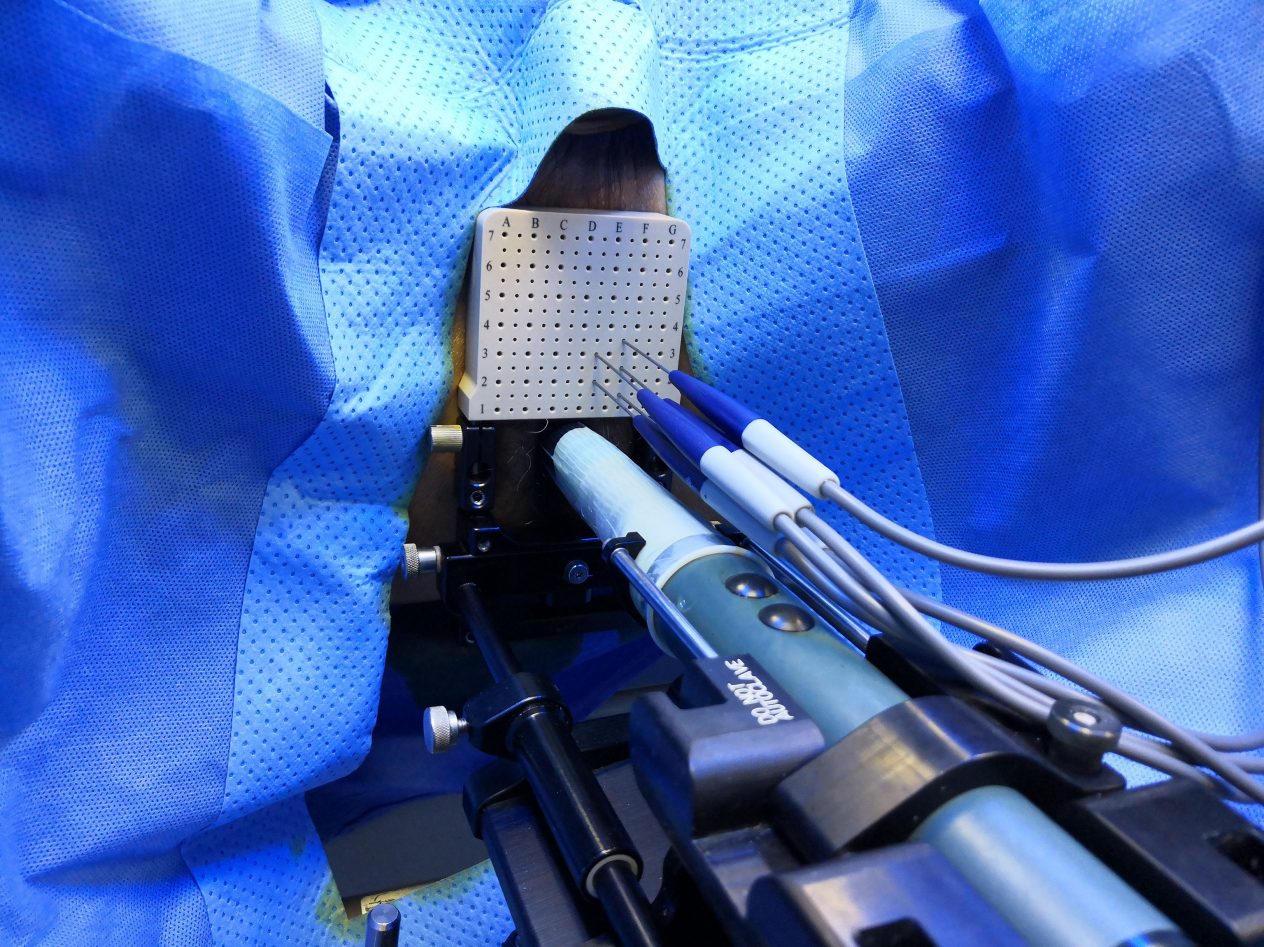
Electrode needles are placed through a brachytherapy template grid and the perineum.

**Figure 5:**
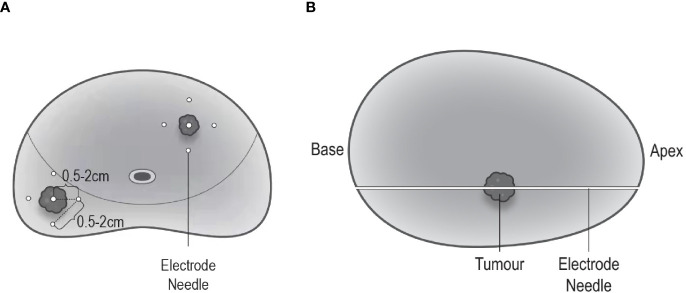
Electrode needle placement. **(A)** Coronal view: one needle is positioned in the center of the targeted lesion, and three to four needles are positioned around the lesion; the distance between each needle ranges 0.5–2.0 cm. **(B)** Sagittal view: maximum electrode exposure length covers the whole length of the prostate (from apex to base).

The ablation area will be planed following an extended ablation strategy (**Figure 2**), which depends on both biopsy and MRI results. The whole procedure of H-FIRE will be performed under general anesthesia with full-muscle paralysis to avoid contractions, employing the composite steep pulse therapeutic apparatus (**Figure 3**) manufactured by REMEDINE company. With the guidance of a biplanar TRUS probe, the electrode needle will be placed to the target lesion by getting through a 5-mm brachytherapy template grid and the perineum (**Figure 4**). The electrode needles will be placed as follows (**Figure 5**): (1) one needle will be placed in the center of the targeted lesion, and three or four needles will be positioned around the lesion; (2) the distance of each needle is between 0.5 and 2.0 cm; and (3) the maximum electrode exposure length ranges from 0.5 to 4.0 cm, in order to penetrate the whole prostate. After placing the needle, the device will be set to deliver predefined pulses for every targeted two needles. The entire procedure is expected to last 40 to 120 min, depending on the total pulse number, which was confirmed by the size and shape of the lesions to be ablated.

### Monitoring

A team of independent clinical research associates (CRA) with all more than 5 years of experience is responsible for being familiar with the trial protocol and monitoring all researchers and all participants involved in the whole process of this trial. The CRA’s role is to (1) monitor the trial plan, the record forms, and the case report form before the start of the trial; (2) monitor participants’ informed consent and enrolment rates; (3) monitor the compliance of participants and investigators with the protocol; (4) monitor the data quality and authenticity; (5) monitor adverse events and serious adverse events; and (6) monitor the device supplied, installed, and reclaimed by relevant regulations.

### Harms and Adverse Events

All harm relevant or not relevant to the procedure of H-FIRE will be recorded, including (1) suspected adverse effects related to the IRE device; (2) obvious non-related diseases that do not appear before the trial; (3) deterioration of existing conditions; (4) accidental injury; (5) adverse events caused by improper use of the device; and (6) any adverse events caused by user error. The adverse events during the trial will be evaluated according to Common Terminology Criteria for Adverse Events (CTCAE), which is widely used as a standard for severity grading scale in clinical trials. The serious adverse events include (1) deaths; (2) fatal illness or injury; (3) permanent defects in body structure or body function; and (4) requirement of hospitalization or prolonged hospitalization. Harms and adverse events will be recorded from the beginning of the trial to 1 month after the biopsy. All AEs and SAEs will be recorded by a CRA team member immediately and reported to the ethics committee and the department of quality supervision and control within 24 h.

### Data Management

The clinical and other trial-related data will be recorded by researchers wholly and carefully and monitored by CRA strictly. All original data will be signed by researchers during the trial and transferred to each center’s clinical research institution for storage. To ensure the accuracy of the data, two data administrators input and proofread the data in double copies independently. After input, data will be verified by both edit check and manual check. The inconsistent data found in the verification will be corrected in time or the discrepancy report issued by the data department should be submitted to the researcher for confirmation before making changes. The confirmed data will be locked and then exported for statistical analysis.

## Discussion and Limitations

The protocol has some potential limitations. First, the prostate biopsy as an endpoint may not be as accurate as the specimen from prostatectomy. However, a prostatectomy may do additional harm or side effects for those who had already undergone an ablation. Besides, a targeted biopsy plus template-guided mapping biopsy has been reported by several studies for its accuracy being sufficiently high for detecting a clinically significant cancer (15–17). Indeed, it is more in line with actual clinical work. Second, the endpoint from an H-FIRE procedure to re-biopsy is only 6 months, making this trial challenging to come to firm conclusions regarding the efficacy and safety of H-FIRE in the long term. However, we will keep following up with the patients as recommended by the international multidisciplinary consensus project (18) when the trial ends. The subsequent results will also be published in peer-reviewed journals.

## Conclusion

This multicenter trial aims to evaluate the efficacy and safety of H-FIRE in treating prostate cancer. Efficacy will be assessed by prostate biopsy 6 months after treatment, while safety will be assessed by adverse events report and questionnaires. This study has been approved by the committee for medical and health ethics of all centers and registered on ClinicalTrials.gov (NCT03838432).

## Patient and Public Involvement

The development of the research question of this trial is based on the patients with low- to intermediate-risk localized prostate cancer for a novel treatment that may lead to fewer side effects and thus increase the quality of life. This trial protocol was written without patient or public involvement. The participants were not involved in the contribution of the design, recruitment, or conduction of the study. Each participant will be informed of the latest results at follow-up and receive a summary of the main finding at the end of the trial.

## Data Availability Statement

The original contributions presented in the study are included in the article/Supplementary Material. Further inquiries can be directed to the corresponding author.

## Ethics Statement

Ethical approval was obtained from the ethics committee of all participating centers. The results of the study will be disseminated and published in international peer-reviewed journals.

## Author Contributions

Conceptualization: H-FW and B-MH. Data curation: H-FW, B-MH, WX, W-GY, LY, B-JD, Z-EZ, H-ZL, YZ, Y-QW, Z-KS, HZ, S-DW, S-CR, XG, L-hW, and C-LX. Formal analysis: H-FW, B-MH, Z-EZ, L-hH, YZ, and L-hW. Investigation: H-FW, B-MH, WX, W-GY, LY, B-JD, Z-EZ, H-ZL, YZ, Y-QW, Z-KS, and HZ. Supervision: XG, L-hW, and C-LX. Original draft: H-FW and B-MH. Review and editing: XG, L-HW, and C-LX. All authors contributed to the article and approved the submitted version.

## Funding

This trial is mainly funded by REMEDINE. REMEDINE is a healthcare company that aims at researching, developing, manufacturing, selling, and renting class III medical devices. This work is also supported by the National Key Research and Development Program of China (No. 2019YFC0119100), Shanghai Pudong New District Health System Medical Talents Training Plan, China (No. PWRd2020-17), fund of development on Science and Technology of Shanghai Pudong New District, China (No. PKX2020-S11), and Shanghai “Action Plan of Technological Innovation” (No. 18441910900). The funder was not involved in the study design, collection, analysis, interpretation of data, the writing of this article or the decision to submit it for publication.

## Conflict of Interest

The authors declare that the research was conducted in the absence of any commercial or financial relationships that could be construed as a potential conflict of interest.

## Publisher’s Note

All claims expressed in this article are solely those of the authors and do not necessarily represent those of their affiliated organizations, or those of the publisher, the editors and the reviewers. Any product that may be evaluated in this article, or claim that may be made by its manufacturer, is not guaranteed or endorsed by the publisher.
